# Is Increased Echogenicity Related to a Decrease in Glomerular Filtration Rate? Objective Measurements in Pediatric Solitary Kidney Patients—A Retrospective Analysis

**DOI:** 10.1371/journal.pone.0133577

**Published:** 2015-08-06

**Authors:** Yong Seung Lee, Mi-Jung Lee, Myung-Joon Kim, Young Jae Im, Sang Woon Kim, Neddy Lee Lim, Sang Won Han

**Affiliations:** 1 Department of Urology and Urological Science Institute, Yonsei University College of Medicine, Seoul, Republic of Korea; 2 Department of Radiology, Yonsei University College of Medicine, Seoul, Republic of Korea; University of Sao Paulo Medical School, BRAZIL

## Abstract

Quantitative measurements of renal echogenicity using a graphic program show close correlation with renal histology in adult patients, but this has neither been applied in pediatric patients nor correlated with glomerular filtration rate (GFR). To determine the direct relationship between echogenicity and GFR, we retrospectively analyzed 91 patients with a solitary functioning kidney under the age of 10, who underwent ultrasonography and serum cystatin C evaluation on a single day between January 2013 and December 2014. Echogenicity was quantified as previously reported. Echogenicity and kidney length were correlated with age-matched values of serum cystatin C-based GFR. Evaluation was performed at a median age of 17.1 months. GFR was low for age in eight of 54 right solitary kidney patients and four of 37 left solitary kidney patients. The right kidney-liver ratio was significantly elevated in the right decreased GFR group, while the left kidney-spleen ratio was not different in the left decreased GFR group. Age-matched longitudinal kidney length ratios were similar between the decreased and normal GFR groups for both sides. This is the first report to objectively prove the relationship between echogenicity and renal function in patients with a right solitary kidney. The right kidney-liver echogenicity ratio, measured objectively, showed feasibility in clinical practice as it showed a close relationship with decreased renal function when increased. However, absolute kidney echogenicity values, or the left kidney-spleen echogenicity ratio, were not independent markers for decreased renal function.

## Introduction

Ultrasonography is the fundamental imaging modality in daily pediatric urological practice. The existence of hydronephrosis, longitudinal kidney length, corticomedullary differentiation, and cortical thickness are all known important parameters in the analysis of pediatric renal ultrasonography.[1] Renal echogenicity is another parameter as its increase is an important sign of renal parenchymal disease.[2, 3] However, it still has limited use, as it is too subjective to quantify. To overcome this issue, Manley and O’Neill first introduced quantitative measurement of renal echogenicity.[4] They scanned ultrasonographic images and measured the echogenicity of the right kidney by adjustment with the adjacent liver using graphic programs. Using a similar method, Moghazi *et al*. reported a close correlation between renal histopathology and echogenicity in adult patients.[5] However, this measurement has neither been correlated with glomerular filtration rate (GFR) nor applied in pediatric patients. To determine the direct relationship between echogenicity and GFR, only solitary kidney patients should be included in the analysis. We have applied this method in pediatric patients with a solitary kidney and correlated it with GFR for the first time in this study.

## Materials and Methods

### Patients

The database of the Department of Pediatric Urology at our institution was retrospectively queried for pediatric patients under the age of 10 years with a solitary functioning kidney. Because this study was performed retrospectively, the Institutional Review Board/Ethics Committee of Severance Hospital approved this study without the need for informed consent (approval number: 4-2014-0944). Patient records were anonymized and de-identified prior to analysis. Among these, 91 patients who underwent ultrasonography and serum cystatin C evaluation on the same day between January 2013 and December 2014 were included in this study. Exclusion criteria included: history of prematurity, grade 2 or higher hydronephrosis graded by the Society for Fetal Urology grading system,[6] known vesicoureteral reflux, the presence of another urinary tract anomaly or glomerular disease in the solitary functioning kidney, coexisting hepato-biliary or spleen disease, and systemic disease.

### Data collection

Data regarding sex, laterality, age at evaluation, glomerular filtration rate, echogenicity of right kidney along with liver or left kidney with spleen, and longitudinal kidney length were collected. The glomerular filtration rate (GFR) was calculated based on the level of serum cystatin C using a previously reported formula by Grubb *et al*.: 84.69 × serum cystatin C (mg/L)^-1.680^ × 1.384 (if child <14 years)[7]. The decreased GFR group was determined using recently reported reference GFR levels in Japanese children using the values of creatinine and cystatin C-based GFR.[8] When the calculated GFR was below the 2.5 percentile level for age, it was considered as ‘decreased GFR’. We additionally calculated cystatin C-based GFR Z-scores by age to analyze the correlation between renal function and echogenicity. The mean and standard deviation values were not shown in the original report; however, in contacting Uemura et al., we received these values and were thus able to calculate the Z-scores (Table 1).

**Table 1:** The reference values of GFR for each age group. The median, 2.5 percentile, and 97.5 percentile values were cited from a report by Uemura et al.[8] The mean and standard deviation values were not shown in their original report; however, in contacting them, we received these values and calculated the Z-scores.

Age	N	2.5% tile	50.0% tile	97.5% tile	Mean	Standard deviation
3–5 months	17	76.6	91.7	106.7	91.7	9.5
6–11 months	47	75.7	98.5	133.0	100.8	15.8
12–17 months	31	83.3	106.3	132.6	106.6	13.7
18 months–16years	1042	83.5	113.1	156.7	115.2	18.3

### Renal ultrasonography and quantification of echogenicity

**Fig 1:**
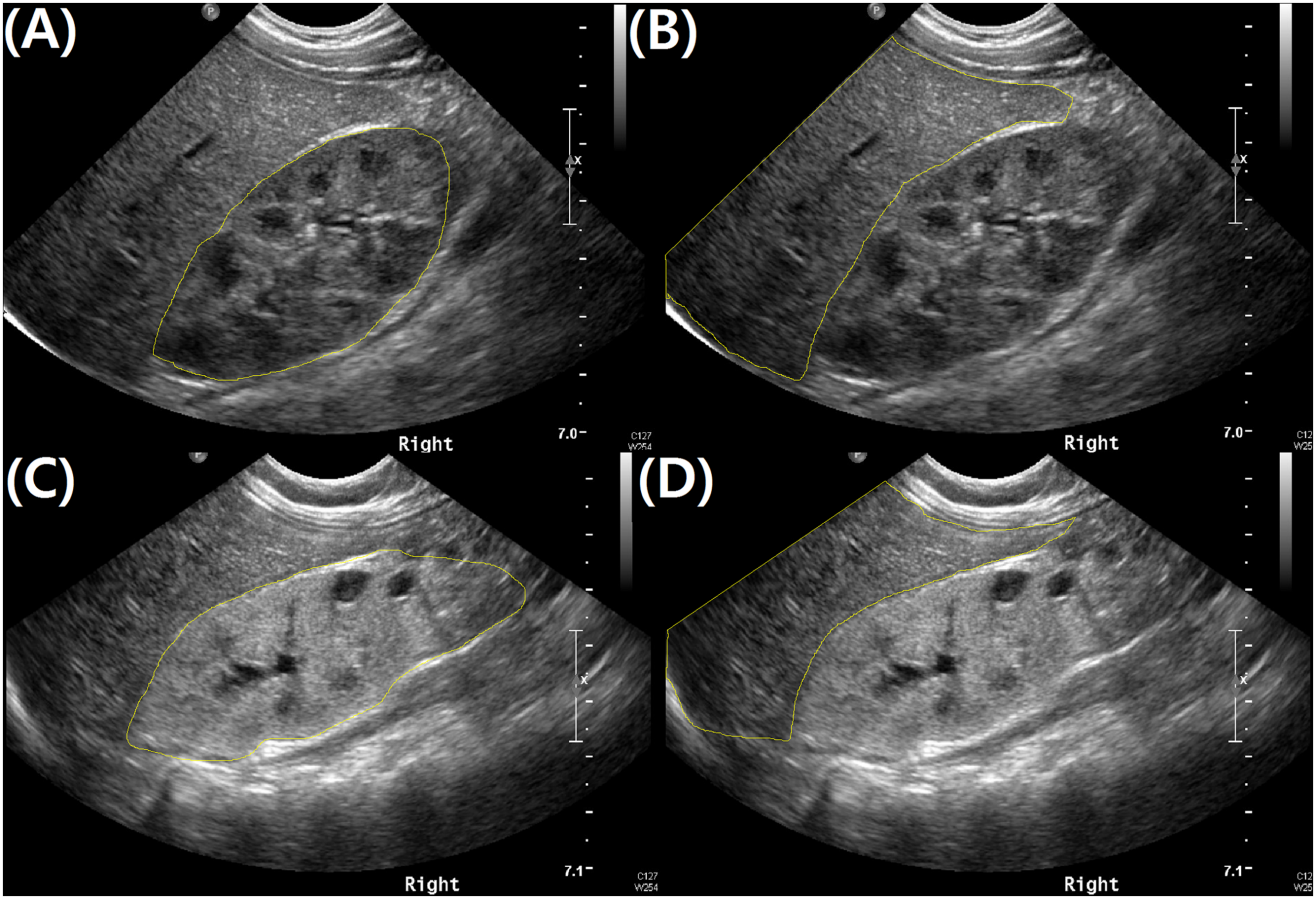
Longitudinal images of the right solitary kidneys with adjacent livers were captured in a 6-month-old female infant with an estimated glomerular filtration rate of 117.2 mL/min/1.73 m^2^ (A), (B) and in a 7-month-old male infant with an estimated glomerular filtration rate of 51.6 mL/min/1.73 m^2^ (C), (D). In each picture, the region of interest was outlined around the entire kidneys (A), (C) and around the livers (B), (D) with ImageJ software, version 1.48v (National Institutes of Health, USA). Renal echogenicity was measured in 256-degree grayscale numerically from 0 to 255. The mean pixel density of the right kidney and liver were 70.3 and 67.3 respectively in the former patient with a right kidney-liver echogenicity ratio of 1.04, and 119.6 and 70.4 respectively in the latter patient with a ratio of 1.70.

All patients were instructed to increase their water intake with their usual diet, without intravenous hydration or diuretics, and ultrasounds were performed after oral hydration according to the previous recommendation of the Society for Fetal Urology.[6] Parents and older children with communication skills were instructed to increase water intake before evaluation for hours. In the case of infants or younger children, parents were instructed to increase the water intake of the patient to the extent possible. Two experienced pediatric radiologists (MJK and MJL) performed all the ultrasonographic evaluations using an iU22 ultrasound unit (Philips Ultrasound, Bothell, WA, USA) with a 5–8 or 1–5 MHz convex transducer. Gain was adjusted for the optimization of imaging by the radiologists during the evaluation. Echogenicity was quantified as previously reported.[4, 5, 9] In brief, among pictures in same study, longitudinal images of the right kidney with the adjacent liver, or the left kidney with the spleen, were chosen and captured (Fig 1). In cases of a left solitary kidney, an image of the liver was additionally captured, although it was not located in the same image as the left kidney. In each picture, the region of interest (ROI) was outlined around the whole kidney and around the liver or spleen with ImageJ software, version 1.48v (National Institutes of Health, USA). Then, renal echogenicity was measured numerically according to the 256 degrees of grayscale labeled by the software, from 0 (black) to 255 (white), for each pixel within the ROI. The inverse ratio of the mean pixel densities of the kidney and adjacent organ (liver in case of the right kidney and spleen in case of the left kidney) was calculated. In cases of a left solitary kidney, the calculation was performed again between the left kidney and the liver. Along with echogenicity, longitudinal kidney length was measured and adjusted with mean length of age-matched normal children, as reported by Kim et al.[10] Echogenicity and kidney length were correlated with age-matched values of serum cystatin C-based GFR. Each sonographic parameter was measured by a single investigator (YSL) to avoid interobserver variability, as in the study by Moghazi *et al*.[5]

### Statistical Analysis

Univariate analyses were performed using the Fisher’s exact test and Mann-Whitney *U*-test in each kidney. Spearman correlation analyses were performed to analyze the eGFR Z-scores for age and the right kidney-liver echogenicity ratio. In addition, to analyze the change in renal echogenicity with age, correlation analysis was also performed between the echogenicity ratio and age in the patients with normal GFR in each kidney group. SPSS software, version 18.0 (SPSS Inc., Chicago, IL) was used. P-values <0.05 were considered statistically significant.

## Results

Fifty-four patients with right solitary functioning kidneys and 37 patients with left solitary functioning kidneys were enrolled in this study (Table 2). Ultrasonography was performed and serum cystatin C evaluated at a median age of 17.1 months. The following were the causes leading to the condition of solitary kidney: multicystic dysplastic kidneys in 82 (90.1%) patients, and renal agenesis in the other 9 (9.9%) patients. The mean pixel density of the right kidney was 68.6 (interquartile range [IQR]: 56.8–82.5), significantly different from that of the left kidney (52.9; IQR: 41.4–76.5; p = 0.005).

**Table 2:** Characteristics of 91 pediatric patients with solitary kidney.

Variable	Number (%), or median (interquartile range)
Gender (male:female)	41:50
Laterality (right:left)	54:37
Cause of non-functioning kidney	
MCDK	82 (90.1)
Renal agenesis	9 (9.9)
Mode of presentation	
Prenatal diagnosis	88 (96.7)
Urinary tract infection	2 (2.2)
Palpable mass	1 (1.1)
Median age at evaluation (months)	17.1 (8.1–27.5)
Existence of proteinuria on urinalysis	0 (0.0)
Serum cystatin C (mg/L)	1.01 (0.90–1.16)
Median glomerular filtration rate (mL/min/1.73m^2^)	115.3 (91.3–139.9)
Decreased glomerular filtration rate for age	12 (13.2)
Median kidney echogenicity	
Right kidney^a^	68.6 (56.8–82.5)
Left kidney^b^	52.9 (41.4–76.5)
Total	65.0 (51.5–79.2)
Median liver echogenicity^a^	63.5 (51.9–77.6)
Median spleen echogenicity^b^	62.3 (47.4–79.5)
Median kidney size	6.8 (6.3–7.9)

MCDK: multicystic dysplastic kidney.

^a^54 patients with right solitary kidney.

^b^37 patients with left solitary kidney.

### Right kidney-liver echogenicity ratio

**Fig 2:**
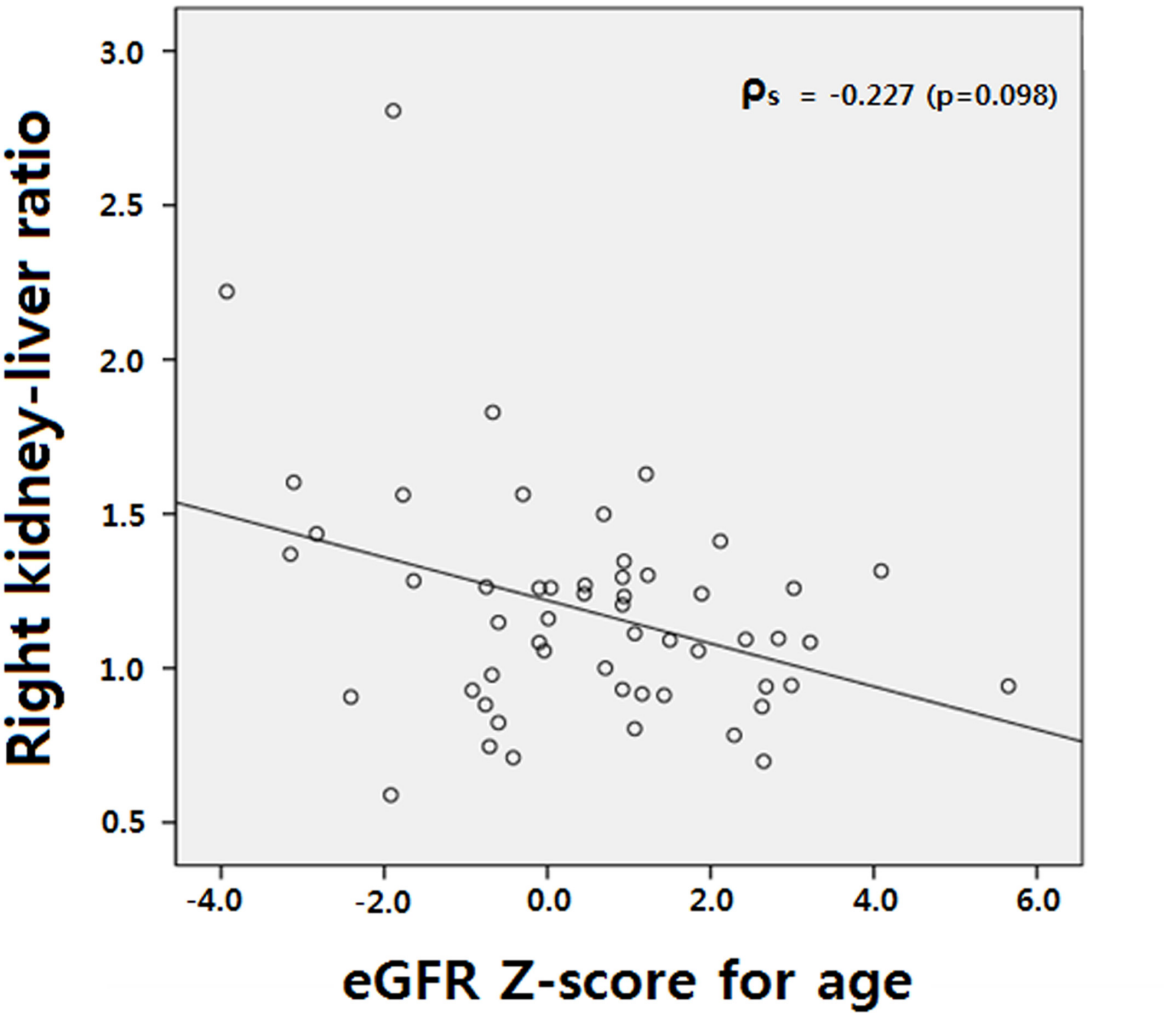
Correlation analysis showed weak correlation between eGFR Z-scores for age and the right kidney-liver echogenicity ratio. The Spearman correlation coefficient was –0.227 (p = 0.098).

Among the 54 patients with right solitary kidneys, GFR was decreased in eight right solitary kidney patients (14.8%) for their age (Table 3). Although the median kidney echogenicity was not different between the groups (p = 0.658), the right kidney-liver ratio was significantly elevated in the decreased GFR group (p = 0.029). The age-matched longitudinal kidney length ratio was not different between the groups (p = 0.422). Correlation analysis showed weak correlation between eGFR Z-scores for age and the right kidney-liver echogenicity ratio. The Spearman correlation coefficient was –0.227 (p = 0.098). A scatter diagram is shown in Fig 2.

**Table 3:** Comparison of clinical parameters between the non-decreased and decreased glomerular filtration rate groups among 54 patients with right solitary kidney.

Variables	Non-decreased (n = 46)	Decreased (n = 8)	p-value
Gender (male:female)	18:28	4:4	0.702
Age at evaluation (months)	16.6 (IQR: 7.5–26.7)	10.5 (IQR: 7.1–23.4)	0.450
Median glomerular filtration rate (mL/min/1.73 m^2^)	119.2 (IQR: 101.4–140.6)	63.2 (IQR: 53.6–78.8)	<0.001
Median kidney echogenicity	68.8 (IQR: 58.0–82.4)	59.5 (IQR: 49.3–109.4)	0.658
Median liver echogenicity	65.4 (IQR: 53.2–77.6)	49.9 (IQR: 31.6–80.0)	0.263
Median kidney-liver echogenicity ratio	1.09 (IQR: 0.93–1.26)	1.50 (IQR: 1.02–2.07)	0.029
Median kidney size	6.9 (IQR: 6.1–7.9)	6.3 (IQR: 5.7–7.2)	0.311
Median kidney size-age matched ratio	1.06 (IQR: 0.93–1.18)	0.99 (IQR: 0.89–1.13)	0.422

IQR: interquartile range.

**Fig 3:**
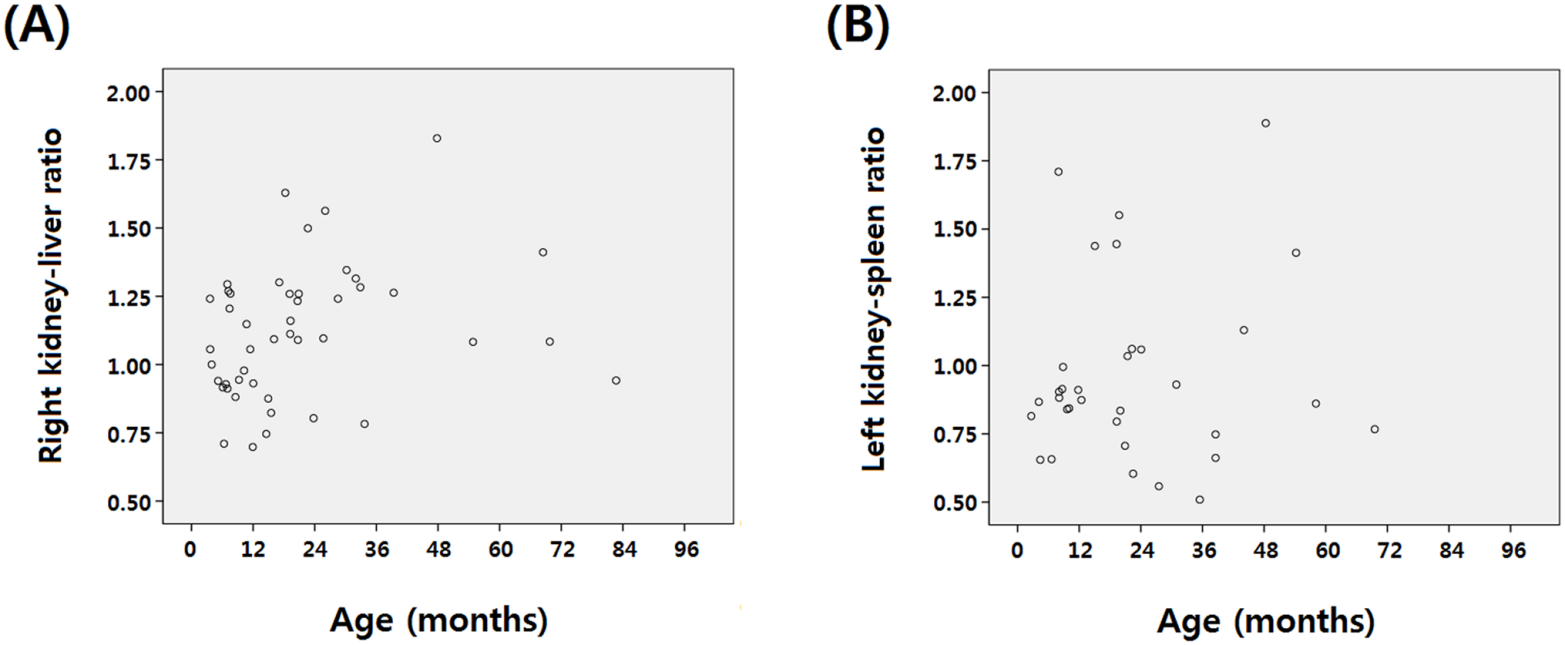
To analyze the change in echogenicity with age, Spearman correlation analysis was performed in the normal glomerular filtration rate group for each kidney. (A) Among 46 patients with right solitary kidneys and a normal glomerular filtration rate, the age and right kidney-liver echogenicity ratio revealed a correlation coefficient of 0.361 with a p-value of 0.014. (B) In the analysis of the correlation between age and left kidney-spleen echogenicity ratio in 33 patients with left solitary kidneys and a normal glomerular filtration rate, the correlation coefficient was 0.015 with a p-value of 0.935.

To analyze the change in renal echogenicity with age, Spearman correlation analysis was performed between the right kidney-liver echogenicity ratio and age in 46 patients with normal GFR. It revealed a correlation coefficient of 0.361 with a p-value of 0.014 (Fig 3A).

### Left kidney-spleen echogenicity ratio

GFR was decreased in four left solitary kidney patients (10.8%) for their age (Table 4). The left kidney-spleen ratio was not different in these four left solitary kidney patients with decreased GFR compared with the left solitary kidney patients with normal GFR (p = 0.493). The left kidney-liver ratio also showed no significant difference between groups (p = 0.114).

**Table 4:** Comparison of clinical parameters between patients with normal and decreased glomerular filtration rates among 37 patients with left solitary kidney.

Variables	Non-decreased (n = 33)	Decreased (n = 4)	p-value
Gender (male:female)	18:15	1:3	0.340
Age at evaluation (months)	19.8 (IQR: 8.8–33.2)	7.0 (IQR: 4.4–29.3)	0.140
Median glomerular filtration rate (mL/min/1.73m^2^)	117.2 (IQR: 97.0–151.0)	72.2 (IQR: 69.3–77.4)	<0.001
Median kidney echogenicity	52.2 (IQR: 41.4–69.2)	83.8 (IQR: 48.9–108.3)	0.092
Median spleen echogenicity	61.4 (IQR: 46.2–77.8)	77.6 (IQR: 56.7–94.7)	0.186
Median liver echogenicity	59.5 (IQR: 44.3–74.0)	56.0 (IQR: 41.2–73.5)	0.906
Median kidney-spleen echogenicity ratio	0.88 (IQR: 0.76–1.06)	0.92 (IQR: 0.80–1.45)	0.493
Median kidney-liver echogenicity ratio	0.90 (IQR: 0.75–1.12)	1.35 (IQR: 0.98–1.98)	0.114
Median kidney size	6.9 (IQR: 6.6–7.9)	6.6 (IQR: 5.8–7.4)	0.285
Median kidney size-age matched ratio	1.05 (IQR: 1.00–1.16)	1.04 (IQR: 0.91–1.13)	0.620

IQR: interquartile range.

Correlation analysis was performed between the left kidney-spleen echogenicity ratio and age in 33 patients with normal GFR. The correlation coefficient was 0.015 with a p-value of 0.935 (Fig 3B).

## Discussion

Manley and O’Neill first introduced quantitative measurement of renal echogenicity in 2001.[4] They scanned ultrasonographic images and measured the echogenicity of the right kidney by adjustment with the liver using graphic programs. Moghazi *et al*. used a similar method and correlated the ultrasonographic findings with renal histological parameters.[5] They revealed that, compared to renal size, cortical thickness, or parenchymal thickness, renal echogenicity showed the strongest correlation with renal histological parameters, such as glomerular sclerosis, tubular atrophy, interstitial fibrosis, and interstitial inflammation. Recently, Hershkovitz *et al*. reported changes in fetal renal echogenicity during the fetal period using similar method.[9] They gathered pictures of the longitudinal kidney from ultrasonography and converted them into 256 degrees of grayscale using a common graphic program. Then they also normalized the difference in gain using liver echogenicity. For this adjustment with the liver, Manley and O’Neill, and Moghazi et al. could analyze right kidneys only. In this study, we used a similar method in pediatric patients with a solitary functioning kidney. In our study, decreased GFR was related to an increased right kidney-liver echogenicity ratio. However, correlation analysis between the eGFR Z-scores and renal echogenicity revealed only a weak correlation. Therefore, it seems that decreased echogenicity does not imply increased renal function, although increased echogenicity is closely correlated with decreased renal function. Previous reports include patients with bilateral kidneys. However, it was not possible to obtain the relationship between echogenicity and GFR for each kidney, as neither the information on the differential renal function of each kidney nor the total GFR was assessed. To overcome this problem, we performed this study including only solitary kidney patients. To our knowledge, this is the first report to objectively prove the relationship between increased echogenicity and decreased renal function.

There have been a few reports of the subjective measurement of renal echogenicity in pediatric patients. Spira *et al*. reported that 83.3% of chronic kidney disease patients with a solitary functioning kidney showed increased echogenicity.[3] Chi *et al*. revealed increased echogenicity to be a poor prognostic factor in pediatric hydronephrosis patients.[2] In addition, it does not always reflect irreversible renal injury: Wiersma *et al*. reported that in children with acute illness, renal echogenicity increases for weeks, even without renal disease.[11] Peerboccus *et al*. reported that a change in echogenicity can be observed, even over the course of a single day, depending on the hydration status.[12] Nevertheless, there has been no report of an objective measurement of renal echogenicity in pediatric patients. Our study is the first report to apply objective measurements of renal echogenicity in pediatric patients.

Just as the echogenicity of the right kidney has been frequently compared with that of the liver, the echogenicity of the left kidney has been compared with the spleen, clinically.[13] Yet, the objectively measured echogenicity of the left kidney adjusted by that of the spleen has not been previously reported. In this study, we analyzed the echogenicity of the left kidney adjusted by that of the spleen. However, unlike our right kidney-liver analysis, we did not observe clinical significance. During the ultrasonographic evaluation, the right kidney is usually covered by the liver while the left kidney is partially covered by spleen. This difference could lead to the difference in gain. Although the grayscale density of the spleen showed similar values as that of liver, those of the right kidney and left kidney were significantly different. This result appears to be due to differences in the echogenicity of the liver and spleen, causing a gain difference, rather than any real differences between the right kidney and left kidney. In addition, as none of the patients in the study had a disease of the liver or spleen, the difference in liver echogenicity between the non-decreased GFR group (65.4) and the decreased GFR group (49.9) in right solitary kidney patients appears to be caused by gain adjustment. Similarly, the difference in spleen echogenicity between the non-decreased GFR group (61.4) and the decreased GFR group (77.6) in the left solitary kidney patients also appears to be caused by gain adjustment. Although it also showed no statistical significance, the left kidney-liver echogenicity ratio was higher in the decreased GFR group. These results may indicate that the liver is more feasible than the spleen as a controlled organ. Using the liver as the reference organ for both kidneys might have produced a different result; however, this option was not available in our retrospective study. Further prospective studies that control for gain would yield useful information on the relationships among the liver, spleen, and kidney.

The echogenicity of the right kidney showed a statistically significant correlation with age. This contradicts a previous report: Han and Bobcock reported that the echogenicity of the right kidney was lower than that of the liver after 3 years of age in normal children.[13] They also revealed the echogenicity of the left kidney to be lower than that of the spleen after 6 months of age. Although there is a difference between the objective measurement in our study and the subjective measurement in Han and Bobcock's study, there might other reasons. First, there could be a difference in hydration status during ultrasonography. Echogenicity in pediatric patients is influenced largely by hydration.[12] After hydration, the echogenicity of kidney increases due to the significant expansion of tubules that then provide two distinct acoustic surfaces.[4] Although we did not use intravenous hydration or diuretics for ultrasonography, patients were instructed to increase water intake before evaluation, which would appear to have greater validity in older children with better communication skills. In addition, the fact that only patients with solitary kidney were included in this study could be another reason, due to the possible effect of compensatory renal growth. In the case of the fetal kidney, the kidney has been known to show different echogenicities according to the trimester.[9] The echogenicity decreased in the second and third trimesters when the collecting system is stable and new nephrons emerge rapidly. In patients with a solitary kidney, compensatory renal growth is observed from the fetal period to 1–2 years of age.[14–16] During this period, echogenicity should decrease, as has been observed during the second and third trimester. This study, performed with solitary kidney patients under the age of 10 years, cannot be taken to represent the renal echogenicity of the general population. As the normal change in renal echogenicity during childhood constitutes important clinical information, further investigation performed with objective measurements in the normal control group should be mandatory.

Nevertheless, the study has limitations. Its retrospective design introduces the potential for selection bias. Although we used a similar method in previous studies, the gain was not controlled due to the retrospective nature, and this could affect the renal echogenicity value among images even in the same study. However, Manley and O’Neill have previously reported a difference of less than 2.8% in renal echogenicity among pictures in the same study.[4] Moreover, the fact that even the images of uncontrolled gain showed clinical significance paradoxically reveals the high feasibility of this method in daily practice. In this study, for each picture, the ROI was outlined around the whole kidney rather than a specific region. Although, Manley and O’Neill first introduced the partial measurement of renal parenchyma,[4] Hershkovitz *et al*. measured the echogenicity of the whole kidney.[9] We chose whole kidney measurement as it is simple and easy, as well as free from selection bias. However, the medulla, renal pelvis, and sinus fat were included in the analysis. To minimize the effect of collecting system inclusion, we excluded patients with hydronephrosis. In addition, sinus fat is known to be less prominent in this age group. However, this could still affect the echogenicity value. Another limitation is that the hydration status was not controlled in all patients. This could lead to an overestimation of cystatin C-based GFR, and also affect the echogenicity of the kidney. Peerboccus *et al*. reported a change in echogenicity after hydration.[12] In this study, however, analysis of the time interval between hydration and ultrasonography was not available. In addition, we used a single measurement of cystatin C to correlate it with the ultrasonography performed on the same day. Although the intra-individual variability of serum cystatin C has been reported to be less than that of serum creatinine in pediatric patients with decreased GFR,[17] serial follow up of both cystatin C and ultrasonography might yield better results.

## Conclusions

This is the first report to objectively prove the relationship between echogenicity and renal function in patients with a right solitary kidney. The right kidney-liver echogenicity ratio, measured objectively, showed feasibility in clinical practice as it showed a close relationship with decreased renal function when increased. Nevertheless, the absolute values of kidney echogenicity alone, or the left kidney-spleen echogenicity ratio, were not independent markers for decreased renal function.
